# Loss of phosphatase and tensin homolog expression castration‐sensitive prostate cancer predicts outcomes in men after prostatectomy

**DOI:** 10.1111/iju.15592

**Published:** 2024-10-01

**Authors:** Yoshinori Yanai, Shuji Mikami, Yota Yasumizu, Toshikazu Takeda, Kazuhiro Matsumoto, Shigehisa Kitano, Mototsugu Oya, Takeo Kosaka

**Affiliations:** ^1^ Department of Urology Keio University School of Medicine Tokyo Japan; ^2^ Department of Diagnostic Pathology Keio University School of Medicine Tokyo Japan; ^3^ Department of Advanced Medical Development, Division of Clinical Chemotherapy The Cancer Institute Hospital of Japanese Foundation for Cancer Research, The Cancer Chemotherapy Center of Japanese Foundation for Cancer Research Tokyo Japan

**Keywords:** biochemical recurrence, phosphatase and tensin homolog, prostate cancer, prostatectomy, tumor suppressor genes

## Abstract

**Objectives:**

This study aimed to investigate the potential for using the phosphatase and tensin homolog (PTEN) gene as a prognostic marker in post‐prostatectomy patients with castration‐sensitive prostate cancer (PCa).

**Methods:**

A total of 180 patients with castration‐sensitive PCa who underwent radical prostatectomy at our institution were included in this study. PTEN expression was evaluated using immunohistochemistry, and patients were classified into two groups based on the staining intensity: PTEN‐Normal and PTEN‐Loss. The association between PTEN expression and biochemical recurrence was analyzed using the Cox proportional hazards model.

**Results:**

Patients in the PTEN‐Loss group had a higher risk of biochemical recurrence (hazard ratio, 4.642; 95% confidence interval, 2.137–10.083; *p* < 0.001) and a lower recurrence‐free rate compared to the PTEN‐Normal group (35% vs. 75%). In addition to clinicopathological factors, such as the serum prostate‐specific antigen level, Gleason score, and T stage, evaluation of PTEN expression improved the prediction of biochemical recurrence after prostatectomy (area under the curve, 0.577 vs. 0.688).

**Conclusions:**

Low PTEN expression is a significant predictor of biochemical recurrence in patients with castration‐sensitive PCa who have already undergone prostatectomy.

**Abbreviations & Acronyms**
AUC: area under the curve
BCR: biochemical recurrence
CSPC: castration‐sensitive prostate cancer
PCa: prostate cancer
PTEN: phosphatase and tensin homolog
ROC: receiver operating characteristic curve
TSGs: tumor suppressor genes

## INTRODUCTION

Increasing evidence suggests that prostate cancer (PCa) is a highly heterogeneous disease,^1^ leading to a wide range of prognoses. Understanding and overcoming intratumor heterogeneity in locally invasive PCa, however, is an unmet medical need in research surrounding PCa. It is difficult to clarify the diversity among prognoses using the existing clinical parameters, which include prostate‐specific antigen (PSA) levels, the Gleason score, and the clinical T stage. Even after prostatectomy, some cases of PCa can be aggressive, lethal, and resistant to androgen deprivation therapy. We previously reported that the addition of tumor immune cell analysis to clinical and pathological parameters is useful in predicting the prognosis of PCa with seminal vesicle invasion after prostatectomy.^2^ In this study, we explored another alternative strategy to clarify the heterogeneity of PCa by adding existing clinical and pathological parameters and evaluated its efficacy in improving prognostic accuracy. In men with metastatic castration‐resistant PCa who showed disease progression while receiving enzalutamide, abiraterone, apalutamide, docetaxel, or olaparib, comprehensive genome profiling was considered to decide the subsequent treatment.^3^ Since the advent of genomic profiling, many studies have demonstrated the effects of tumor suppressor genes (TSGs) in patients with advanced metastatic PCa.^4^, ^5^, ^6^, ^7^ However, comprehensive genome profiling is not usually performed in patients with castration‐sensitive prostate cancer (CSPC). This study aimed to highlight the significance of TSG immunohistochemistry in patients with CSPC.

Phosphatase and tensin homolog (*PTEN*) is one of the representative TSGs and plays an important role in regulating cell growth and division. The *PTEN* gene encodes a tumor suppressor protein that is widely expressed and has both lipid phosphatase and protein phosphatase activities. Its most well‐known catalytic function is to remove the 3′‐phosphate from phosphoinositide‐binding protein 3, converting it to phosphatidylinositol diphosphate. This action counteracts the phosphatidylinositol‐3 kinase signaling pathway. This leads to the inhibition of downstream targets like Akt, the mammalian target of rapamycin, and S6 kinase, which play roles in preventing apoptosis and promoting cell proliferation and migration.^8^, ^9^, ^10^ The loss of function or deletion of *PTEN* is relatively frequent, as observed in approximately 40% of Caucasian men with PCa.^11^, ^12^ The incidence of *PTEN* loss in Asian men with PCa is lower (14%) compared to that in Caucasian men.^13^, ^14^ However, there are no reports on the stratification of CSPC according to PTEN expression at the protein level after prostatectomy in Japan. This study, therefore, aimed to clarify the potential use of PTEN immunohistochemistry as a prognostic marker in post‐prostatectomy patients with CSPC.

## METHODS

### Patients

We retrospectively searched the medical records of patients who underwent radical prostatectomy between January 2010 and December 2018 at our institution and monitored post‐prostatectomy serum PSA levels in each patient. Biochemical recurrence (BCR) was diagnosed by detecting an increase in two successive post‐prostatectomy PSA levels (>0.2 ng/mL) without clinical or radiographic evidence of disease.^15^ We excluded patients with missing data, those who received adjuvant therapy, and those without a nadir post‐prostatectomy PSA level <0.2 ng/mL. After applying the exclusion criteria, a total of 180 patients were enrolled in this study. The protocol for this study was approved by the Institutional Review Board of Keio University Hospital (#20160084), and written informed consent was obtained from all study participants. The study was performed in accordance with the Declaration of Helsinki.

### Immunohistochemistry

The pathological diagnoses were obtained from the patient's medical records, specifically the pathology report of the specimen obtained during prostatectomy. All prostate specimens were formalin‐fixed and paraffin‐embedded after excision. Immunohistochemistry was performed using an anti‐PTEN antibody (#9559, 1:150 dilution; Cell Signaling, Danvers, MA, USA).

### Evaluation of immunostaining

All sections were scanned using a NanoZoomer‐XR high‐resolution digital slide scanner (C12000; Hamamatsu Photonics, Hamamatsu, Shizuoka, Japan). Three authors (YY, TK, and MS), blinded to the patients' clinical data, independently evaluated immunoreactivity by counting the stained nuclei and cytoplasm. The average number of cells counted by each three investigators was used for analysis. Counts with <20% PTEN cells were categorized as PTEN‐Loss, while all other counts were categorized as PTEN‐Normal.

### Statistical analysis

Differences in continuous variables between the two groups were evaluated using the Mann–Whitney *U* test, while the chi‐squared test was used to analyze differences in the number of patients between the two groups. Univariate and multivariate analyses were performed to identify predictive factors for BCR using the Cox proportional hazards model with stepwise forward selection. Kaplan–Meier curves were constructed to evaluate postoperative BCR‐free survival. PTEN expression and clinicopathological factors were used to predict BCR using receiver operating characteristic (ROC) curves and the area under the ROC curve (AUC). Data were reported as medians (interquartile range; IQR). All reported *p*‐values were two‐sided, and statistical significance was set at *p* < 0.05. Statistical analyses were performed using SPSS statistical software (version 27.0; https://www.ibm.com/analytics/spss‐statistics‐software).

## RESULTS

### Patient characteristics

The clinical characteristics of the patients are summarized in Table 1. The median follow‐up period was 5.8 (IQR 4.8–8.7) years. Among the 180 patients, the median age at the time of surgery was 66 (IQR 61–70) years, the median PSA value at the time of diagnosis was 8.6 (5.9–13.1) ng/mL, and the median prostate volume upon excision was 29.0 (20.9–39.0) mL. A total of 156 patients were categorized as having PTEN‐Normal expression, compared to 24 with PTEN‐Loss. There were no significant differences in the follow‐up period, age, prostate volume, clinical T stage, pathological T stage, pathological N stage, Gleason score, or surgical margin status based on PTEN expression (*p* = 0.850, 0.801, 0.571, 0.766, 0.110, 0.732, 0.224 or 0.204, respectively). In contrast, serum PSA level was significantly higher and BCR was significantly more common in the PTEN‐Loss group (*p* = 0.003 and <0.001, respectively).

**TABLE 1:** Patients clinical characteristics according to PTEN expression.

	Total	PTEN‐Normal	PTEN‐Loss	*p*‐Value
Patients (*n*)	180	156	24	
Median follow‐up, year (IQR)	5.8 (4.8–7.7)	5.8 (4.8–7.5)	5.7 (4.2–9.0)	0.850
Median age at diagnosis, year (IQR)	66 (61–70)	66 (61–70)	67 (61–71)	0.801
Median PSA at diagnosis, ng/mL (IQR)	8.6 (5.9–13.1)	8.1 (5.7–11.4)	11.9 (9.4–19.8)	0.003
Median prostate volume, mL (IQR)	29.0 (20.9–39.0)	28.7 (20.8–38.5)	30.0 (21.0–45.2)	0.571
Clinical T stage				0.766
T1		22	3	
T2		111	17	
T3–4		23	4	
Pathological T stage				0.110
T2		110	13	
T3–4		46	11	
Pathological N stage				0.732
Negative		155	24	
Positive		1	0	
Gleason grade group				0.224
1,2,3		112	20	
4,5		44	4	
Surgical margin status				0.204
Negative		93	11	
Positive		63	13	
Biochemical recurrence				<0.001
No		117	8	
Yes		39	16	

Abbreviations: IQR, interquartile range; PSA, prostate‐specific antigen; PTEN, phosphatase and tensin homolog.

### Distribution and prognostic impact of PTEN expression

**FIGURE 1:**
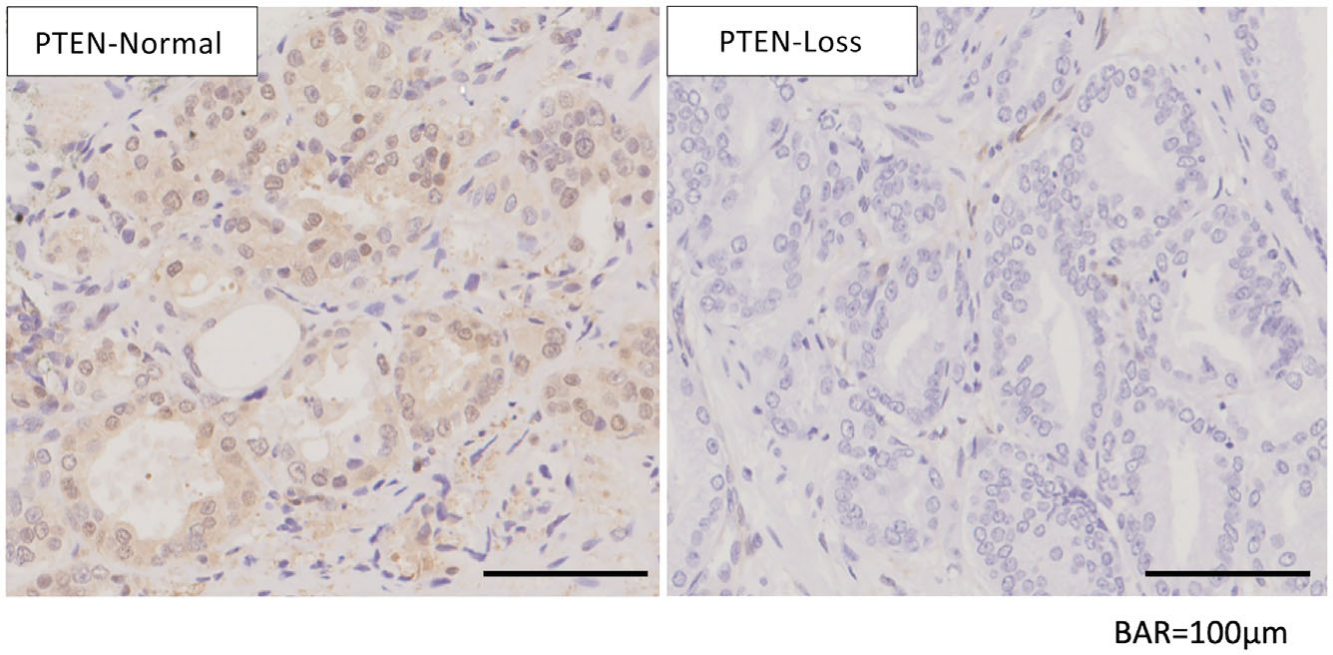
Phosphatase and tensin homolog (PTEN) immunostaining of prostate cancer after prostatectomy.

**FIGURE 2:**
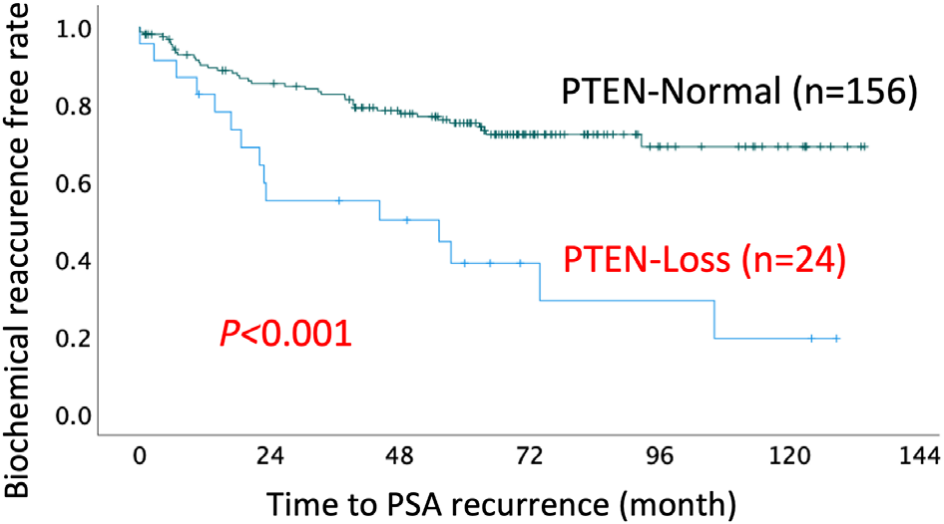
Kaplan–Meier estimates of phosphatase and tensin homolog (PTEN) expression. PSA, prostate‐specific antigen.

**FIGURE 3:**
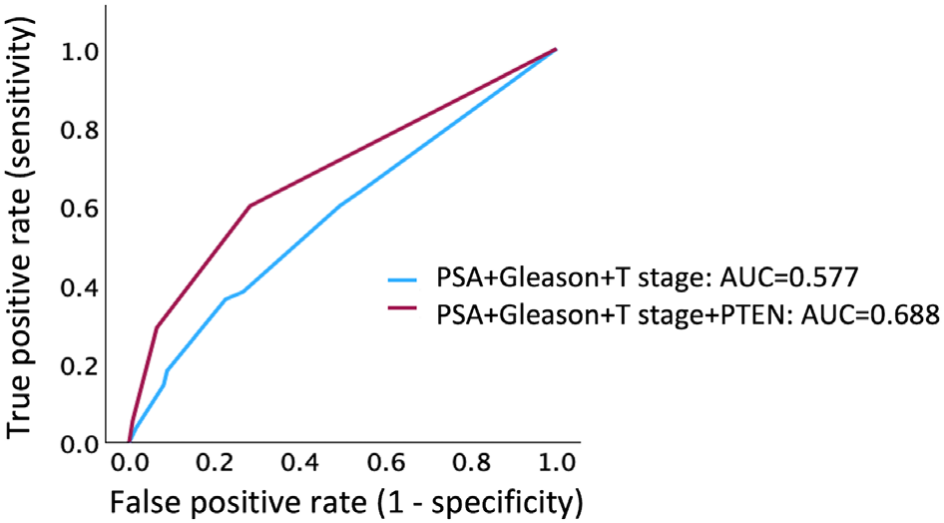
Receiver operating characteristics curves for prediction of biochemical recurrence using clinicopathological factors or phosphatase and tensin homolog (PTEN) expression. AUC, area under the curve; PSA, prostate‐specific antigen.

Representative immunohistochemical images are shown in Figure 1, showing that PTEN expression was observed in the cytoplasm. Table 2 shows the results of the univariate and multivariate analyses of the association between PTEN expression and BCR, which showed a statistically significant difference between the PTEN‐Normal and PTEN‐Loss groups (*p* < 0.001). Loss of PTEN expression was associated with BCR, with an estimated hazard ratio for a 20% cutoff value for PTEN of 4.642 (95% confidence interval, 2.137–10.083; *p* < 0.001) in the multiple logistic regression analysis. The BCR‐free rates of the PTEN‐Normal and PTEN‐Loss groups were 75% and 35%, respectively (*p* < 0.001; Figure 2). The AUC for predicting post‐prostatectomy BCR based on clinicopathological factors such as serum PSA level, Gleason score, and clinical T stage was 0.577; however, when PTEN expression was added to these factors, the AUC was elevated to 0.688 (Figure 3). As seen in Table S1, we determined the cutoff value based on the proportion of PTEN observed through immunohistochemistry. The most significant results were observed at 15% and 20%, with a hazard ratio of 6.435; therefore, for this study, we set the cut‐off value at 20%.

**TABLE 2:** Univariable and multivariable analysis of the association of PTEN expression with biochemical recurrence.

Parameters	Univariate	Multivariate	
		HR (95% CI)	*p*‐Value
Age (years)	0.348		
PSA at diagnosis (ng/mL)	0.063		
Prostate volume (mL)	0.225		
Clinical T stage			
T1–2 versus T3–4	0.187		
Pathological T stage			
T2 versus T3–4	0.003		
Pathological N stage			
Negative versus positive	0.514		
Gleason grade group			
1,2,3 versus 4,5	0.022		
Surgical margin status			
Negative versus positive	0.001	4.026 (1.810–8.954)	<0.001
PTEN expression			
Normal versus loss	<0.001	4.642 (2.137–10.083)	<0.001

CI, confidence interval; HR, hazard ratio; PSA, prostate‐specific antigen; PTEN, phosphatase and tensin homolog.

## DISCUSSION

The results of this study suggested that lower PTEN expression, as detected by immunohistochemistry, plays a significant role in predicting BCR outcomes in post‐prostatectomy patients with PCa. Copy number loss in the *PTEN* gene is sometimes observed in PCa, resulting in the loss of PTEN protein expression and activation of protein kinase B signaling pathways. Activation of the PI3K/Akt pathway by PTEN loss enhances androgen receptor signaling and facilitates the survival of androgen‐independent PCa cells.^16^, ^17^, ^18^ Hence, the loss of PTEN expression has been linked to the progression and metastasis of PCa, as well as drug resistance to androgen receptor signal inhibitors, taxanes, and radiation therapy.^19^, ^20^, ^21^, ^22^ Furthermore, the loss of PTEN expression in patients with PCa has been associated with an increased risk of cancer recurrence.^23^, ^24^ Therefore, we expect that using PTEN loss as a biomarker for prognosis prediction will enable personalized treatment and optimization targeting PTEN loss.

Previous studies have demonstrated that patients with CSPC with low *PTEN* expression have lower response rates to androgen deprivation therapy, whereas the loss of *RB1* gene expression is associated with an increased risk of developing castration‐resistant PCa along with worse overall survival.^22^, ^23^, ^24^, ^25^ Comprehensive genome profiling, however, is not usually performed in patients with CSPC. In this study, we used immunohistochemistry, a technique to detect and visualize protein in tissue samples, to assess PTEN expression in post‐prostatectomy patients with CSPC. In Western PCa patients, the frequency of *PTEN* loss is high (approximately 40%) and is particularly prominent in advanced cancers and high‐risk groups. In contrast, the frequency of *PTEN* loss in Japanese patients is reported to be low (approximately 14%).^11^, ^12^, ^13^, ^14^ This may be related to differences in the disease patterns associated with *PTEN* loss by race and ethnicity. The fact that *PTEN* deficiency occurs in more Western patients may be related to the fact that other genetic abnormalities that contribute to PCa progression (e.g., *TP53* and *RB1* mutations) are found at higher rates in Western patients. In contrast, in Japanese patients, genetic abnormalities other than *PTEN* may act as the primary progression factor. Differences in diet and obesity rates may also influence gene mutation patterns in PCa. High‐fat diets and obesity in Western countries may influence tumor progression associated with *PTEN* deficiency. On the other hand, low‐fat diets are more popular in Japan, which may influence the lower frequency of *PTEN* deficiency and the rate of PCa progression. That is why it is highly significant to examine the feasibility of using PTEN immunohistochemistry in a Japanese cohort as in this study.

We determined the cutoff value as 20% based on the proportion of PTEN observed through immunohistochemistry (Table S1). Some previous reports have used a cutoff value of 50% for the proportion of stained cells in PTEN immunostaining,^26^, ^27^ while others have used 0% to evaluate the complete loss of PTEN expression.^28^, ^29^ Therefore, we calculated sequentially with varying values and set the cutoff at 20% in this study. Notably, this value might change with an increase in the number of study cases, which is one of the limitations of this study. Contrarily, most previous studies have focused on Caucasians.^30^ Since PCa progression and characteristics can differ by race and ethnicity, establishing a cutoff value in this study with Japanese patients is particularly valuable.

In this study, we used Cell Signaling's PTEN antibody because it has been used in other studies at our institution. However, the specificity and sensitivity of different antibodies are different, and it is another limitation of this study to examine whether other antibodies will show the same results as this study.

This study has other limitations as well, including the small number of cases and difficulty performing genetic testing in patients after radical prostatectomy. There may also be differences in the frequency and combination of genetic mutations other than *PTEN* between Japanese and Western countries, and further investigations are needed on other TSGs, including *RB1* and *TP53*, which are our future research topics. The authors emphasize, however, that PTEN immunohistochemistry can be a valuable prognostic marker in early‐stage PCa, particularly in cases with limited comprehensive genomic profiling.

## AUTHOR CONTRIBUTIONS

**Yoshinori Yanai:** Formal analysis; data curation; investigation; writing – original draft. **Shuji Mikami:** Data curation; investigation. **Yota Yasumizu:** Data curation; writing – review and editing. **Toshikazu Takeda:** Data curation; writing – review and editing. **Kazuhiro Matsumoto:** Data curation; writing – review and editing. **Shigehisa Kitano:** Validation; writing – review and editing. **Mototsugu Oya:** Validation; writing – review and editing. **Takeo Kosaka:** Conceptualization; writing – original draft; supervision.

## CONFLICT OF INTEREST STATEMENT

Kazuhiro Matsumoto is an Editorial Board Member of the *International Journal of Urology* and a co‐author of this article. To minimize bias, they were excluded from all editorial decision‐making related to the acceptance of this article for publication.

## APPROVAL OF THE RESEARCH PROTOCOL BY AN INSTITUTIONAL REVIEWER BOARD

The protocol for this study was approved by the Institutional Review Board of Keio University Hospital (#20160084). The study was performed in accordance with the Declaration of Helsinki.

## INFORMED CONSENT

Written informed consent was obtained from all of the study participants.

## REGISTRY AND THE REGISTRATION NO. OF THE STUDY/TRIAL

Not applicable.

## ANIMAL STUDIES

Not applicable.

## Supporting information


Table S1.
