# Supplementary material for: Loss of phosphatase and tensin homolog expression castration‐sensitive prostate cancer predicts outcomes in men after prostatectomy
Source: Int J Urol. 2024 Oct 1;32(1):39–44. doi: 10.1111/iju.15592 (PMC11730643; doi:10.1111/iju.15592)
Supplement: Supplementary file 1 — Table S1. [file IJU-32-39-s001.docx]

**Table S1. The cutoff value based on the proportion of *PTEN* observed through immunohistochemistry**

| PTEN cut-off | Patients (biochemical recurrence) | Univariate | Multivariate | |
| --- | --- | --- | --- | --- |
|  |  |  | HR (95% CI) | P-value |
| 5% | Loss (4) vs Normal (24) | 0.058 | 3.755 (0.840 - 16.779) | 0.083 |
| 10% | Loss (7) vs Normal (21) | 0.008 | 6.047 (1.869 - 19.572) | 0.003 |
| 15% | Loss (8) vs Normal (20) | 0.005 | 6.435 (2.098 - 19.736) | 0.001 |
| 20% | Loss (8) vs Normal (20) | 0.005 | 6.435 (2.098 - 19.736) | 0.001 |
| 25% | Loss (8) vs Normal (20) | 0.011 | 5.374 (1.739 - 16.606) | 0.003 |
| 30% | Loss (8) vs Normal (20) | 0.021 | 5.374 (1.739 - 16.606) | 0.003 |
| 40% | Loss (8) vs Normal (20) | 0.088 | 3.107 (1.063 - 9.079) | 0.038 |
| 50% | Loss (8) vs Normal (20) | 0.128 | 2.781 (0.947 - 8.166) | 0.063 |
| 60% | Loss (9) vs Normal (19) | 0.085 | 3.718 (1.313 - 10.528) | 0.013 |
| 70% | Loss (11) vs Normal (17) | 0.224 | 2.515 (0.919 - 6.882) | 0.072 |
| 80% | Loss (18) vs Normal (10) | 0.061 | 3.115 (1.071 - 9.057) | 0.037 |
| 90% | Loss (24) vs Normal (4) | 0.981 | NA | NA |
